# Pancreatic Resections in Renal Failure Patients: Is It Worth the Risk?

**DOI:** 10.1155/2014/938251

**Published:** 2014-02-09

**Authors:** K. S. Norman, S. R. Domingo, L. L. Wong

**Affiliations:** ^1^Department of Surgery, University of Hawaii John A. Burns School of Medicine, Honolulu, HI 96813, USA; ^2^Queens Medical Center, Honolulu, HI 96813, USA; ^3^University of Hawaii Cancer Center, Honolulu, HI 96813, USA

## Abstract

*Background*. Chronic kidney disease affects 20 million US patients, with nearly 600,000 on dialysis. Long-term survival is limited and the risk of complex pancreatic surgery in this group is questionable. Previous studies are limited to case reports and small case series and a large database may help determine the true risk of pancreatic surgery in this population. *Methods*. The American College of Surgeons National Surgical Quality Improvement Program database was queried (2005–2011) for patients who underwent pancreatic resection. Renal failure was defined as the clinical condition associated with rapid, steadily increasing azotemia (rise in BUN) and increasing creatinine above 3 mg/dL. Operative trends and short-term outcomes were reviewed for those with and without renal failure (RF). *Results*. In 18,533 patients, 28 had RF. There was no difference in wound infections, neurologic or cardiovascular complications. Compared to non-RF patients, those with RF had more unplanned intubation (OR 4.89, 95% CI 1.85–12.89), bleeding requiring transfusion (OR 3.12, 95% CI 1.37–14.21), septic shock (OR 8.86, 95% CI 3.75–20.91), higher 30-day mortality (21.4% versus 2.3%, *P* < 0.001) and longer hospital stay (23 versus 12 days, *P* < 0.001). *Conclusions*. RF patients have much higher morbidity and mortality after pancreatic resections and surgeons should consider this before proceeding.

## 1. Introduction

Although the incidence of end stage renal disease (ESRD) has remained relatively stable in the United States, the prevalence has increased, such that 593,000 people are currently living on hemodialysis, on peritoneal dialysis, or with a functioning kidney transplant. Improvements in renal-replacement therapy, overall treatment, and access to care have increased patient survival in ESRD. In the prevalent population, mortality rates have declined by nearly 25 percent over the last two decades. Patients are living longer on renal replacement therapy and the population currently on dialysis is increasing in age. The overall incidence of dialysis patients is 340 per million persons but since 2000, the adjusted incidence rate of ESRD has increased by 12.2% for those patients 75 years or older (1773 per million persons) [1, 2].

Because of the rising number of aging dialysis patients, there will likely be an increased need for use of hospital resources and cancer care in this population. Multiple studies have consistently showed increased complications and mortality in renal patients undergoing general, vascular, and cardiac surgical procedures [3–8]. Patient selection and vigilant management are recommended in this population.

The question of whether patients with renal failure should undergo major cancer surgery has not been specifically addressed in the medical literature. The complexity of the surgery and overall prognosis of the type of cancer involved will need to be considered in choosing options for these patients. Few studies address outcomes of pancreatic surgery in patients with renal dysfunction and all were single center studies consisting of small series of patients [9–11]. The purpose of this study is to utilize a large, nationwide database to determine outcomes of pancreatic resection in patients with renal failure to assist surgeons and oncologists in selecting the optimal patients for these procedures.

## 2. Materials and Methods

Data for this study was obtained from the American College of Surgeons National Surgical Quality Improvement Program (ACS NSQIP). ACS NSQIP is a prospective, multiinstitutional, and clinical registry created by the Veterans Health Administration in 1994 for quality improvement purposes. Over 130 preoperative variables through 30-day postoperative variables are collected on randomly assigned patients, including patient demographics, surgical profile, preoperative risk assessment, laboratory values, operative information, and 30-day morbidity and mortality rates. A highly trained Surgical Clinical Reviewer (SCR) collects the data. All reviewers receive extensive initial training prior to starting data collection and ongoing training via continuing education. ACS NSQIP monitors accrual rates and data sampling methodologies and conducts audits on a random basis, ensuring highly reliable data [12].

ACS NSQIP participant files for the years 2005–2011 were reviewed and Current Procedure Terminology (CPT) codes were used to identify all patients who underwent pancreatic procedures (48100-48999). We excluded pancreatic biopsy and pancreatic debridement as these were presumably done for unresectable pancreatic malignancies or necrotizing pancreatitis. From the remaining pancreatic cases, two groups were created: patients who had pancreatic resection with renal failure (group RF) and those who underwent pancreatic resection with normal renal function (group non-RF).

Renal failure was defined as an increased creatinine above 3 mg/dL on laboratory studies within 24 hours prior to surgery and patients on dialysis. Dialysis dependent patients were those with acute or chronic renal failure requiring peritoneal dialysis, hemodialysis, hemofiltration, hemodiafiltration, or ultrafiltration within two weeks prior to surgery.

Patient demographics included sex, age, smoking, and alcohol use. The comorbidities considered were diabetes, chronic obstructive pulmonary disease (COPD), congestive heart failure (CHF), hypertension requiring medications, disseminated cancer, and transfusions within 3 days prior to surgery. Postoperative complications of interest were superficial surgical site infection, deep incisional surgical site infection, organ space surgical site infection, wound disruption, pneumonia, urinary tract infections, unplanned intubation, pulmonary embolism, deep vein thrombosis, cardiac arrest requiring cardiopulmonary resuscitation, myocardial infarction, intraoperative or postoperative transfusions, sepsis, and septic shock.

Finally other outcome measures reviewed included operative time, return to the operating room, hospital length of stay, 30-day mortality, and time from operation to death in those patients who expired.

### 2.1. Statistical Analysis

RF versus non-RF groups were compared for baseline characteristics. Numerical values were compared using Student *t*-test. Categorical and dichotomous variables were compared using chi-square testing. Associated risks were expressed as odds ratios (OR) with a 95% confidence interval (CI). All reported *P* values are two-tailed, and for all tests, *P* < 0.05 was considered statistically significant.

With nominal regression, we used preoperative variables of sex, age > 65 years, history of diabetes, smoking, congestive heart failure, use of steroids, albumin < 3.0 gm/dL, bilirubin > 2.0 mg/dL, AST > 90 U/L, hematocrit < 30%, platelet  count < 200 × 10^3^/cc, and prothrombin time > 14 seconds and the presence of renal failure to determine if any of these factors were associated with 30-day mortality.

## 3. Results

During the 7-year period, 2005–2011, there were 18,533 patients who underwent pancreatic resection. Males to females were 8922 to 9585 and mean age was 61.8 years. Of this entire cohort, 28 patients were identified as having renal failure. Specific procedures included partial removal of pancreas (13), pancreatectomy (6), pancreatectomy with pancreatojejunotomy (3), distal pancreatectomy (3), and proximal pancreatectomy (3).

Patient demographics and comorbidities are as listed in Table 1. RF patients were older and were more likely to have diabetes, myocardial infarction within the previous 6 months, hypertension requiring medications, use of steroids, bleeding disorder, disseminated cancer, and a blood transfusion prior to surgery.

Preoperative laboratory studies are as shown in Table 2. RF patients had significantly worse initial bleeding parameters with higher protime and partial thromboplastin time, as well as a lower platelet count. Initial WBC and hematocrit were also lower in RF patients. RF patients had similar bilirubin but higher AST, alkaline phosphatase, and lower albumin prior to pancreas surgery.

Postoperative complications and outcome are detailed in Tables 3 and 4. Most notably, RF patients were more likely to need reintubation, have a cardiac arrest, and have bleeding that required blood transfusion. Although RF patients were not any more likely to have surgical site infections, wound infections, or sepsis, they were more likely to have septic shock. RF patients were more likely to require a return to the operating room. Hospital length of stay and 30-day mortality were significantly higher in RF patients. Of all patients who died in this study, those with RF expired in a mean of 7.8 days compared to 13.3 days in non-RF patients.

Using nominal regression, the factors predictive of 30 day mortality included age 65 or higher, presence of diabetes, history of CHF, steroid use, albumin < 3.0 gm/dL, bilirubin > 2.0 mg/L, Hct < 30%, protime > 14 seconds, and renal failure (see Table 5). The presence of RF had the highest odds ratio at 6.13.

## 4. Discussion

Advances in renal replacement therapy and better management of diabetes and cardiovascular problems have allowed patients with RF to live longer with their chronic illnesses, but they now may be living long enough to develop neoplasms that require surgical intervention. While previous large studies have addressed surgical procedures in dialysis patients, less is known about how these RF patients fare in pancreatic resections.

Previous studies have demonstrated increased surgical morbidity and mortality in patients with renal failure who are dialysis dependent. In the largest study, Gajdos et al. used NSQIP to investigate the effect of long-term dialysis on general surgical procedures. The 1506 dialysis patients had 12.7% mortality (30-day) compared to 1.5% in the 164,094 patients with normal renal function. Dialysis patients also had a higher pulmonary complications, reoperations, cardiovascular complications, surgical site infections, and hospital length of stay. While this study had a large number of dialysis patients, it included a variety of general surgical procedures with varying levels of complexity and is done for both benign and malignant reasons [7].

Pancreatic surgeries are among the most complex of the abdominal procedures. Many factors contribute to this risk but the effect of renal dysfunction has been variably reported. Age has been the most commonly cited risk factor for outcome in pancreatic surgery [13–16]. Diabetes, the reason for pancreatic resection (benign versus malignant), hospital volume, preoperative biliary drainage, and resection of other organs have all been mentioned as contributing to prognosis [9, 11, 17–19]. Renal failure and dysfunction have been shown to contribute to poor outcome in pancreatic head resections in 3 single center studies, but these studies had 300 cases or fewer in each series [9–11].

Two studies attempted to combine multiple risk factors in developing tools to assess patients' risk for pancreatic surgery. Are et al. using the Nationwide Inpatient Sample (NIS) database developed a nomogram to predict outcome for patients undergoing pancreatic resection for malignancy. Renal failure was the factor with the highest number points assigned in the nomogram [20]. On the other hand, Parikh et al. using NSQIP developed a risk calculator to predict outcome after pancreatic resection. Renal failure was not mentioned as a risk factor, but American Society of Anesthesiologists (ASA) classification, functional health status, sepsis, surgical extent, age, dyspnea, body mass index, coronary heart disease, gender, and bleeding disorder were the most important [21].

Two additional studies reviewed outcomes after pancreatic surgery utilizing the NIS and discussed the effect of renal failure. McPhee analyzed in-hospital mortality in patients who underwent pancreatectomy for neoplasm between 1998 and 2003. Of 39,463 patients, 395 (1%) had renal failure and mortality rate of RF patients was 36.1% compared to the overall mortality rate of 5.9% [22]. Teh et al. evaluated the NIS database for major pancreatic resections done for benign and malignant disease between the years of 1988 and 2003. Among the cohort of 103,222 patients, 1% had renal insufficiency and this patient subset had increased in-hospital mortality (OR 6.3), perioperative complications (OR 2.3), and increased mortality following a major complication (OR 3.5) [23]. While these studies are helpful in stratifying renal failure as a risk, the clinical variables and specific outcomes in these RF patients are not described in detail.

Our study clearly demonstrates that renal failure increased morbidity and mortality for patients undergoing pancreatic resection. Although only a cohort of 28 patients, 21% of these patients died in a mean of 7.8 days after surgery. Renal failure increased the 30-day mortality by 6-fold. Patients with renal failure also had a significantly higher rate of postoperative complications with an increased risk of unplanned reintubation, cardiac arrest, septic shock, and need for transfusion. Those patients who did survive remained hospitalized for almost twice as long as patients without RF.

This study did have limitations, as it utilized an administrative database and can only be as precise as the trained staff who enter the data. Although the number of pancreatic resections was large, the number of cases with RF was relative small and the data may have diminished accuracy especially in subcategories and with some of the laboratory values. In addition, NSQIP is able to define a preoperative creatinine but cannot tell us if creatinine is due to a chronic kidney disease or if this is an acute or relatively recent renal dysfunction. One might assume that a patient with acute progressive renal failure would not be subjected to a major pancreatic resection, but unfortunately this study cannot provide us with the exact information. The grouping of the procedures by NSQIP based on procedural codes was quite nonspecific and there was likely variability in coding by the staff. It is likely that “partial removal of pancreas” may have included both distal pancreatic resections and pancreaticoduodenectomies with varying extents of each procedure. A major limitation of the study is that NSQIP database did not report the exact pathology of the resected specimen or the details of the operative procedure. It is unclear if some of these cases represented benign or premalignant lesions such as cystic tumors or neuroendocrine tumors which may have significantly less risk and better short, and long-term outcome. Finally, although NSQIP can identify general complications, it is not a database that can delineate procedure-specific complications so important complications such as pancreatic leaks and fistulas could not be assessed.

## 5. Conclusions

In conclusion, this study establishes a significant negative effect of renal failure on outcome in pancreatic resections. It will be difficult to acquire larger cohorts of patients with this particular problem and NSQIP is really not able to assess risk based on specific diagnoses requiring pancreatic resection. Each RF patient should be assessed for overall risk and especially in the context of expected long-term survival based on the specific pancreatic diagnosis. Those patients who opt for pancreatic resection should be optimized in terms of bleeding parameters and should be monitored carefully for respiratory, bleeding, and septic complications. Strong consideration should be made for nonoperative therapies or no treatment in those patients who have prohibitive risk or limited long-term survival.

## Figures and Tables

**Table 1:** Patient characteristics.

	Total (*n* = 18533)	Group RF (*n* = 28)	Group non-RF (*n* = 18505)	*P* value
Mean age (SD)	61.8 (13.9)	67.9 (13.5)	61.8 (13.9)	*P* = 0.02
Age 65 or older (%)	8659 (46.7%)	16 (57.1%)	8627 (46.6%)	NS (*P* = 0.34)
Males (%)	8922 (48.1%)	17 (60.7%)	8905 (48.1%)	NS (*P* = 0.41)
Diabetes	4197 (22.6%)	11 (39.3%)	4186 (22.6%)	*P* = 0.040
History of smoking	4065 (21.9%)	6 (21.4%)	4059 (21.9%)	NS (*P* = 0.95)
History of alcohol use	478 (2.6%)	0	478 (2.6%)	NS (P = 0.45)
History of COPD	815 (4.4%)	3 (10.7%)	812 (4.4%)	NS (*P* = 0.12)
Myocardial infarction within 6 mo.	56 (0.3%)	3 (10.7%)	53 (0.3%)	*P* < 0.0001
Hypertension requiring medications	9620 (51.9%)	24 (85.7%)	9596 (51.9%)	*P* < 0.0001
Congestive heart failure	51 (0.3%)	0	51 (0.3%)	NS (*P* = 0.86)
Transient ischemic attack	338 (1/8%)	1 (3.6%)	337 (1.8%)	NS (*P* = 0.55)
Cerebrovascular disease	255 (1.4%)	1 (3.6%)	254 (1.4%)	NS (*P* = 0.43)
Currently on steroids	383 (2.1%)	3 (10.7%)	380 (2.1%)	*P* = 0.02
Bleeding disorder	507 (2.7%)	6 (21.4%)	501 (2/7%)	*P* < 0.0001
Disseminated cancer	617 (3.3%)	6 (21.4%)	611 (3.3%)	*P* < 0.0001
Prior radiation therapy	376 (2.0%)	0	376 (2.0%)	NS (*P* = 0.48)
Prior chemotherapy	350 (1.9%)	2 (7.1%)	348 (1.9%)	NS (*P* = 0.09)
Transfusion before surgery	149 (0.8%)	3 (10.7%)	146 (0.8%)	*P* = 0.001

**Table 2:** Preoperative laboratory studies. All values with standard deviation.

	Group RF (*n* = 28)	Group non-RF (*n* = 18505)	*P* value
Sodium (mmol/L)	138.1 (3.8)	138.9 (3.2)	NS (*P* = 0.187)
BUN (mg/dL)	45.2 (22.0)	14.8 (7.3)	*P* < 0.0001
Creatinine (mg/dL)	4.0 (2.03)	0.94 (0.5)	*P* < 0.0001
Albumin (gm/dL)	3.0 (0.9)	3.8 (0.7)	*P* < 0.0001
Total bilirubin (mg/dL)	2.0 (3.19)	1.57 (2.51)	NS (*P* = 0.42)
Alanine aminotransferase (U/L)	92.4 (145.0)	49.7 (67.7)	*P* = 0.002
Alkaline phosphatase (U/L)	234.3 (266.3)	162.4 (161.0)	*P* = 0.026
WBC (×108/L)	10.3 (7.23)	7.4 (2.9)	*P* < 0.0001
Hematocrit (%)	30.8 (5.6)	38.0 (5.1)	*P* < 0.0001
Platelet count (×10^3^/mL)	218.5 (107.9)	265.4 (97.0)	*P* = 0.012
Partial thromboplastin time (seconds)	33.2 (9.6)	29.6 (5.8)	*P* = 0.004
Prothrombin time (seconds)	14.8 (3.9)	12.6 (2.4)	*P* < 0.0001

**Table 3:** Postoperative complications.

	Total (*n* = 18533)	Group RF (*n* = 28)	Group non-RF (*n* = 18505)	*P* value
Superficial/skin infection	1524 (8.2%)	1 (3.6%)	1523 (8.2%)	NS (*P* = 0.37)
Deep surgical site infection	361 (1.9%)	1 (3.6%)	360 (1.0%)	NS (*P* = 0.53)
Intra-abdominal infection	1887(10.2%)	2 (7.1%)	1885 (10.2%)	NS (*P* = 0.60)
Wound dehiscence	271 (1.5%)	0	271 (1.5%)	NS (*P* = 0.52)
Postoperative pneumonia	888 (4.8%)	1 (3.6%)	887 (4.8%)	NS (*P* = 0.76)
Need for reintubation	793 (4.3%)	5 (17.9%)	788 (4.3%)	*P* < 0.0001
Pulmonary embolism	210 (1/1%)	0	210 (1.1%)	NS (*P* = 0.57)
Urinary tract infection	934 (5.0%)	1 (3.6%)	933 (5.0%)	NS (*P* = 0.72)
Myocardial infarction	115 (0.6%)	0	115 (0.6%)	NS (*P* = 0.68)
Cardiac arrest	204 (1.1%)	3 (10.7%)	201 (1.1%)	*P* < 0.0001
Cerebrovascular accident	50 (0.3%)	0	50 (0.3%)	NS (*P* = 0.78)
Deep venous thrombosis	416 (2.2%)	2 (7.1%)	414 (2.2%)	NS (*P* = 0.08)
Bleeding requiring transfusion	2108 (11.4%)	8 (28.6%)	2100 (11.3%)	*P* = 0.004
Sepsis	1854 (10.0%)	1 (3.6%)	1853 (10.0%)	NS (*P* = 0.26)
Septic shock	678 (3.7%)	7 (25.0%)	671 (3.7%)	*P* < 0.0001
Return to OR	1141 (6.2%)	4 (14.3%)	1137 (6.2%)	NS (*P* = 0.08)

**Table 4:** Outcome measures.

	Group RF (*n* = 28)	Group non-RF (*n* = 18505)	*P* value
Operative time in minutes (SD)	316 (139.7)	324 (142.7)	NS (*P* = 0.913)
Hospital length of stay in days (SD)	23 (25.7)	12.0 (11.0)	*P* < 0.001
Days from operation to death (SD)	7.83 (7.36)	13.3 (8.54)	NS (*P* = 0.36)
30-day mortality	6/28 (21.4%)	425/18505 (2.3%)	*P* < 0.001

**Table 5:** Predictors of 30-day mortality.

	Odds ratio (95% CI)	*P* value
Renal failure	6.13 (2.32–16.18)	*P* < 0.001
History of congestive heart failure	4.72 (2.13–10.50)	*P* < 0.001
Age 65 or higher	2.44 (1.98–3.01)	*P* < 0.001
Albumin < 3.0 gm/dL	1.94 (1.47–2.56)	*P* < 0.001
History of steroid use	1.93 (1.18–3.16)	*P* = 0.009
Hematocrit < 30%	1.69 (1.24–2.28)	*P* = 0.001
Protime > 14 seconds	1.59 (1.25–2.02)	*P* < 0.001
Diabetes	1.33 (1.08–1.64)	*P* = 0.008
Bilirubin > 2.0 mmol/L	1.02 (0.79–1.32)	NS (*P* = 0.87)
